# New Insight on FGFR3-Related Chondrodysplasias Molecular Physiopathology Revealed by Human Chondrocyte Gene Expression Profiling

**DOI:** 10.1371/journal.pone.0007633

**Published:** 2009-10-29

**Authors:** Laurent Schibler, Linda Gibbs, Catherine Benoist-Lasselin, Charles Decraene, Jelena Martinovic, Philippe Loget, Anne-Lise Delezoide, Marie Gonzales, Arnold Munnich, Jean-Philippe Jais, Laurence Legeai-Mallet

**Affiliations:** 1 Unité U781, Institut National de la Santé et de la Recherche Médicale, Université Paris Descartes-Hôpital Necker, Paris, France; 2 Unité Mixte de Recherche 1313, Institut National de la Recherche Agronomique, Jouy-en-Josas, France; 3 4Clinics, Waterloo, Belgique; 4 Département de Transfert, Institut Curie, Paris, France; 5 Service de Fœtopathologie, Hôpital Necker, Université Paris Descartes, Paris, France; 6 Centre Pluridisciplinaire de Diagnostic Prénatal de Rennes, Hôpital de Rennes, Rennes, France; 7 Service de Biologie du développement, Hôpital Robert Debré, Université Paris Diderot, Paris, France; 8 Service de Génétique et d'Embryologie Médicales, Hôpital Armand Trousseau, Université Pierre et Marie Curie, Paris, France; 9 Service de Biostatistique et Informatique Médicale, Hôpital Necker, Université Paris Descartes, Paris, France; University of California Davis, United States of America

## Abstract

Endochondral ossification is the process by which the appendicular skeleton, facial bones, vertebrae and medial clavicles are formed and relies on the tight control of chondrocyte maturation. Fibroblast growth factor receptor (FGFR)3 plays a role in bone development and maintenance and belongs to a family of proteins which differ in their ligand affinities and tissue distribution. Activating mutations of the FGFR3 gene lead to craniosynostosis and multiple types of skeletal dysplasia with varying degrees of severity: thanatophoric dysplasia (TD), achondroplasia and hypochondroplasia. Despite progress in the characterization of FGFR3-mediated regulation of cartilage development, many aspects remain unclear. The aim and the novelty of our study was to examine whole gene expression differences occurring in primary human chondrocytes isolated from normal cartilage or pathological cartilage from TD-affected fetuses, using Affymetrix technology. The phenotype of the primary cells was confirmed by the high expression of chondrocytic markers. Altered expression of genes associated with many cellular processes was observed, including cell growth and proliferation, cell cycle, cell adhesion, cell motility, metabolic pathways, signal transduction, cell cycle process and cell signaling. Most of the cell cycle process genes were down-regulated and consisted of genes involved in cell cycle progression, DNA biosynthesis, spindle dynamics and cytokinesis. About eight percent of all modulated genes were found to impact extracellular matrix (ECM) structure and turnover, especially glycosaminoglycan (GAG) and proteoglycan biosynthesis and sulfation. Altogether, the gene expression analyses provide new insight into the consequences of FGFR3 mutations in cell cycle regulation, onset of pre-hypertrophic differentiation and concomitant metabolism changes. Moreover, impaired motility and ECM properties may also provide clues about growth plate disorganization. These results also suggest that many signaling pathways may be directly or indirectly altered by FGFR3 and confirm the crucial role of FGFR3 in the control of growth plate development.

## Introduction

Endochondral ossification, the process by which the appendicular skeleton, facial bones, vertebrae and medial clavicles are formed, relies on a tightly controlled chondrocyte maturation process, characterized by successive changes in cell morphology and gene expression. Maturation of chondrocytes leads to the formation of the growth plate, which consists of three main zones containing resting, proliferative or hypertrophic chondrocytes. In order for endochondral ossification to occur, precise temporal and spatial coordination between the different factors providing both positive and negative signals at each step of the process is essential. These factors can act in a synergistic manner, or form negative feedback loops and may participate in signaling pathways such as parathyroid hormone related peptide (PTHrP), Indian hedgehog (IHH), C-type natriuretic peptide (CNP), bone morphogenetic protein (BMP), Wnt and fibroblast growth factor receptor (FGFR) pathways [1]–[3]. Nitric oxide, hypoxia and their downstream effectors have also been shown to act as major key regulators of endochondral ossification [4]–[8]. In addition, cell adhesion pathways, cytoskeleton structure and dynamics and intraflagellar transport have emerged as crucial regulators of both proliferation and hypertrophy [9]–[15]. Intracellular pathways triggered by these signals, as well as their targets are still poorly defined and only a few downstream transcription factors have been studied including the SOX, RUNX, AP1 and the CREB/ATF families [16], [17] as well as Nkx3.2 [18], Snail1 [19] and BAG-1 [20].

The importance of FGFR3 in early bone development was established from the association of point mutations in *FGFR3* with dominant chondrodysplasias ranging in severity from mild (hypochondroplasia [HCH] and achondroplasia [ACH]) to severe (thanatophoric dysplasia [TD]), [21], [22]. Two TD subtypes have been defined based on the clinical presentation: type I (TDI), characterized by marked shortness and bowing of the long bones and type II (TDII), characterized by straight femurs and a moderate to severe cloverleaf skull deformity. Histopathology of the growth plate reveals a disruption of endochondral ossification.

It is generally accepted that FGFR3 is a negative regulator of bone growth and that constitutive activation of FGFR3 signaling is the cause of these disorders [23], [24]. Several signaling pathways have been shown to be affected by FGFR3 activation including STAT1/3 [25]–[27], STAT5 [28], MEK1 [29] and ERK1/2 [30], [31]. Different *in vitro* stimulation studies performed on rat chondrosarcoma (RCS) and primary chondrocytes using FGFs revealed decreased cell proliferation due to cell cycle arrest at G1, up-regulation of c-jun, junD, cyclin-D1, NF-KB, STAT1/3 and p21, activation of members of the pRb family, inactivation of Id1, cyclin-E-Cdk2 complex and reduced AKT phosphorylation [26], [32]–[35]. A two step mechanism leading to cell cycle arrest has also been proposed based on a time course microarray analysis of FGF stimulated RCS cells [36]. The first "growth arrest initiation" step includes down-regulation of key cell cycle genes and some positive regulators of proliferation, and the second "growth arrest maintenance" step is characterized by Cdk inhibition, up-regulation of p21, Rb and p130 dephosphorylation and down-regulation of additional cell cycle protein genes [36]. Taken together, these data support a model in which excessive FGFR3 signaling in chondrocytes reduces bone growth by inhibiting chondrocyte proliferation and some aspects of differentiation. Despite progress in characterization of the FGFR3-mediated regulation of cartilage, many aspects of this regulation remain unclear and downstream events are poorly understood.

The aim of the present study was to examine whole gene expression changes in human pathological cartilage of *FGFR3*-related chondrodysplasias. Control and TDI primary human chondrocyte mRNA expression levels were compared using Affymetrix technology. The analysis of genes associated with cell functions provides new insight into consequences of *FGFR3* mutations on cell cycle regulation, onset of pre-hypertrophic differentiation, concomitant metabolism changes and adhesion. These results also suggest that many signaling pathways may be affected by *FGFR3* and confirm the crucial role of *FGFR3* in skeletal disease.

## Results

### Sample collection

The study was performed using two batches of human chondrocyte primary cultures derived from seven TDI and four control fetuses without skeletal pathology, aged 18 to 25 weeks. The radiological features of TDI such as narrow trunk, curved femurs and platyspondyly were observed in all pathological fetuses. The chondrocytic phenotype of the primary cells was assessed before performing the microarray experiment. Cultured cells retained chondrocytic morphology and a high expression of cartilage specific genes such as *aggrecan*, *collagen type II* compared to *collagen type I* and *SOX9* was observed by RT-PCR (data not shown). Four heterozygous Arg248Cys and three Tyr373Cys mutations were identified in the *FGFR3* extracellular domain by genomic DNA sequencing of TD primary chondrocytes. The control primary chondrocytes did not contain mutations in the FGFR3 domains (Supplementary data S1).

### In silico analysis

#### The transcriptome of the normal chondrocyte

**Figure 1:**
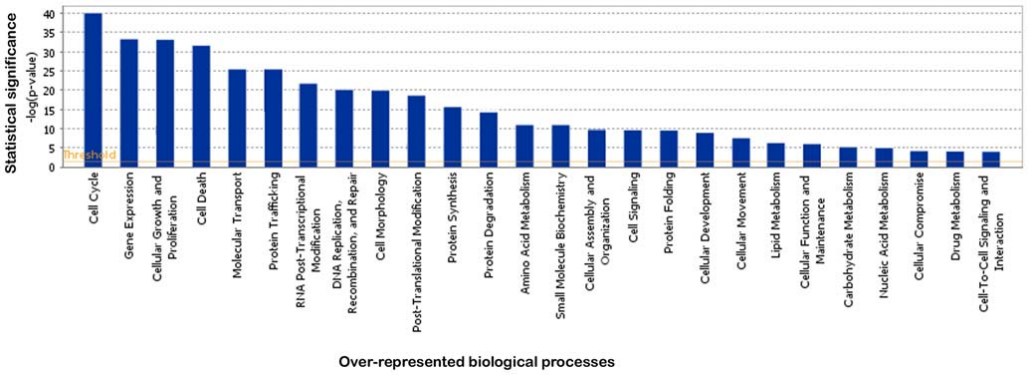
IPA analysis of biological processes associated with expressed genes in the normal chondrocyte. The classical IPA bar chart displays biological functions along the x-axis. The y-axis displays the - (log) significance. Functions are listed from most significant (higher bars) to least significant (lower bars) and the orange horizontal line denotes the threshold for significance (p-value of 0.05).

After data normalization, intensity signals above background (median log_2_ signal intensity above 4) could be detected for 22000 probe sets in human control chondrocytes (supplementary data S2). These probe sets were found to match 11777 known genes, of which 7505 were eligible for functional annotation using Ingenuity Pathway Analysis (IPA). Identified biological functions included cell cycle (859 genes, p-value = 9 10^−41^), gene expression (1231 genes, p-value = 6 10^−34^), cellular growth and proliferation (1188 genes, p-value = 8 10^−34^) and cell death (1658 genes, p-value = 8 10^−32^). Details are given in Figure 1 and supplementary data S3.

**Figure 2:**
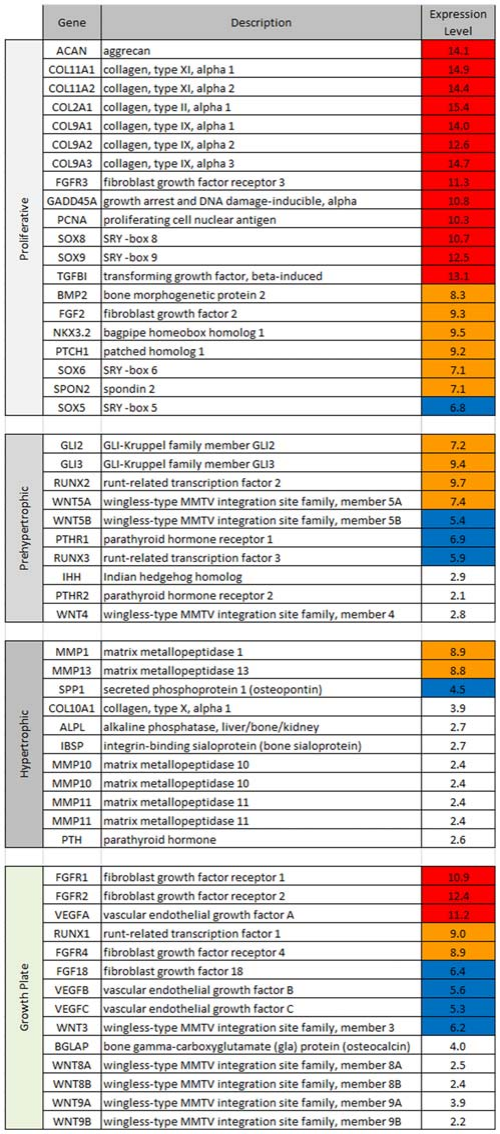
Expression level of cartilage maturation stage specific marker genes in the normal chondrocyte. Genes were classified according to their role in particular stages of cartilage maturation. Their Log_2_ intensity expression values are shown on the right, with high expression values (red), mid values (orange) and low values (blue). Expression values below 4 are considered as background (white). Most expressed genes belong to the proliferative group, suggesting that primary chondrocytes were mainly in a proliferative state.

Expression values of cartilage specific markers were compiled in an attempt to characterize the phenotype of the primary chondrocytes. Most of the genes characteristic of the proliferative zone were highly expressed, including *FGFR3*, *GADD45A* and *SOX8*. Other key genes controlling chondrocyte proliferation such as *NKX3.2*, *SOX9* and *BMP2* were also observed. Major components of the extra-cellular matrix such as *collagen type II, IX* or *XI*, *aggrecan* and *glypican* were likewise highly expressed. Pre-hypertrophic markers, like *Gli* or *RUNX2* were expressed. In contrast, no expression of hypertrophic markers such as *collagen type X*, *alkaline phosphatase*, *IHH*, *PTHrP*, or *PTH* was observed (Figure 2).

#### Biological process modulated by FGFR3 constitutive activation

A two way ANOVA was performed to identify 516 probe sets differentially expressed between TDI and normal chondrocytes, with at least a 50% change in expression level. These probe sets matched to 427 known genes in IPA, of which 291 were eligible for functional annotation. Up regulated genes (265) were as frequent as down-regulated ones (251), with 27 genes showing a fold change greater than 5-fold (supplementary data S4).

**Figure 3:**
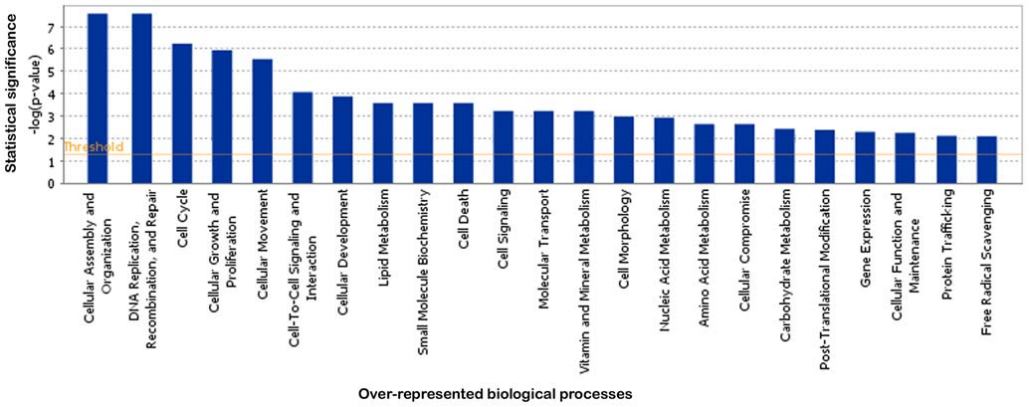
IPA analysis of biological processes associated with modulated genes in TDI chondrocytes. Biological functions associated with significantly modulated genes are shown along the x axis of the bar graph and the - (log) significance along the y-axis. Functions are listed from most significant (higher bars) to least significant (lower bars). The threshold for significance (p-value of 0.05) is shown as an orange horizontal line.

Functional annotation using IPA and FatiGO+ showed that modulated genes were involved in multiple biological processes. All functions usually considered to be affected by *FGFR3* mutations were represented: cell cycle (65 genes), cellular growth and proliferation (111 genes), cell death (75 genes) and cell signaling (35 genes). This analysis also revealed differences in the expression of genes involved in metabolism (lipid, nucleic acid and carbohydrate: 63 genes), in cell-cell interaction (67 genes), cell adhesion (33 genes) and cell motility (51 genes) processes (Figure 3 and supplementary data S4).

**Figure 4:**
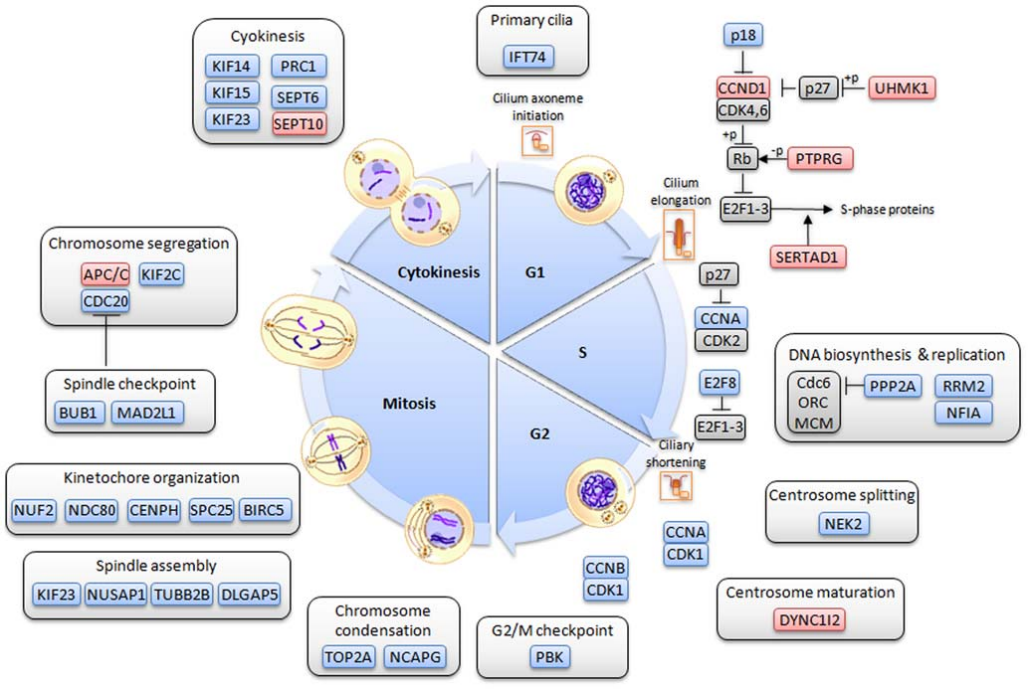
Normal v. TDI chondrocytes: modulated genes and cell cycle. Modulated genes involved in different cell cycle processes are depicted as colored boxes along a diagram showing the five main stages (G1, S, G2, M and cytokinesis). Up-regulated genes are colored in red whereas down-regulated genes are colored in blue. Some key regulator genes, which are not modulated by FGFR3, are colored in grey.

Most of the “cell cycle process” genes which were were down-regulated were also involved in cell cycle progression and checkpoints (e.g. *BUB1, CDC2*, *CDKN2C*, *CCNA1*, *CCNB2, E2F8*), DNA biosynthesis and replication (e.g. *NFIA*, *PPP2A*, *RRM2*), spindle and kinetochore assembly (e.g. *BIRC5*, *CENPH*, *KIF23*, *NDC80*, *TUBB2B*) chromosome segregation and cytokinesis (e.g. *CDC20*, *KIF2C*, *KIF14*, *KIF15*, *KIF23*, *SEPT6*). Conversely, genes promoting G1 progression, especially *cyclin D1* and *UHMK1* were up-regulated whereas *p18*, an inhibitor of G1 progression was down-regulated. Details are given in Figure 4.

**Figure 5:**
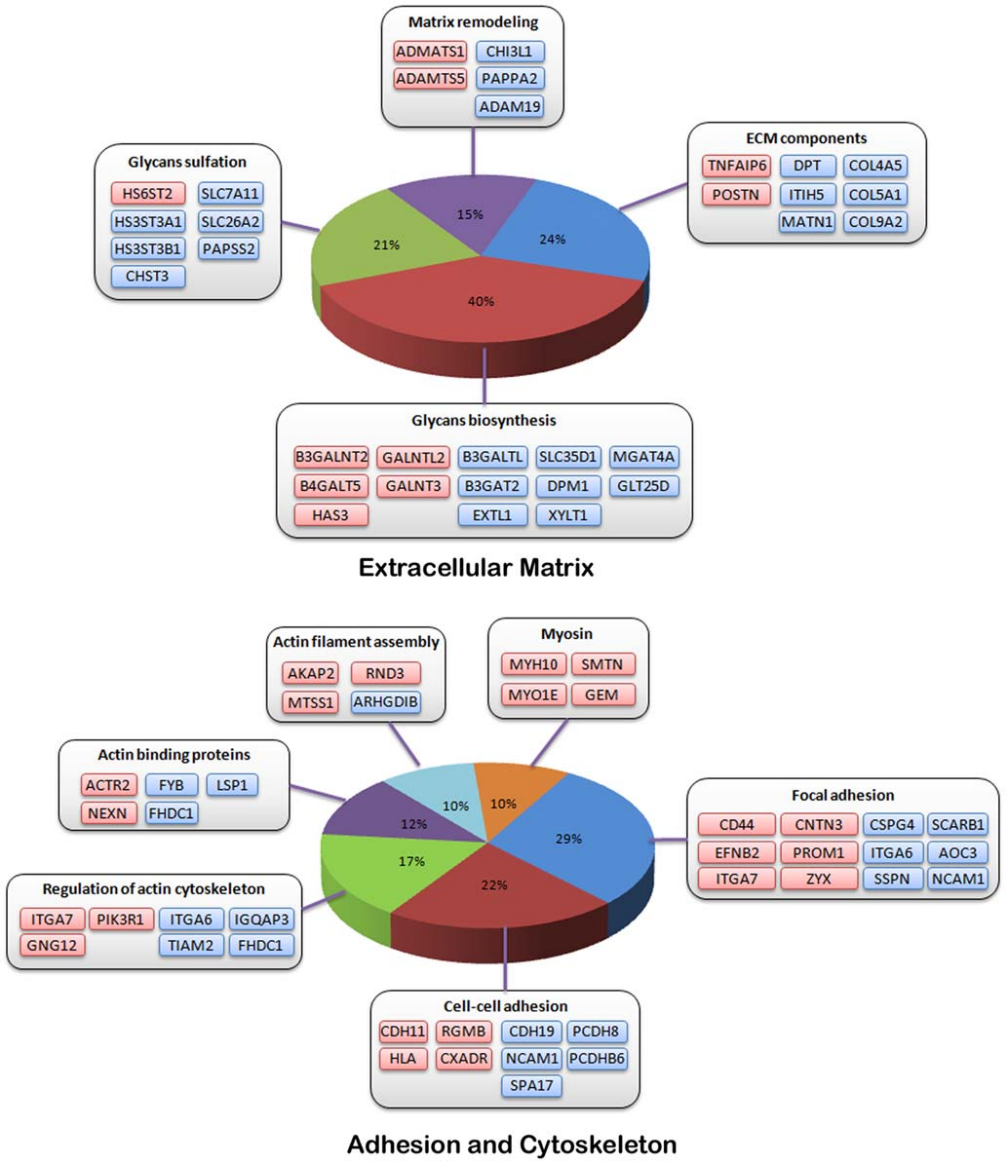
Normal v. TDI chondrocytes: modulated genes, ECM biosynthesis, cytoskeleton and adhesion. Modulated genes are grouped according to their role in these different biological processes. Up-regulated genes are colored in red whereas down-regulated genes are colored in blue.

In addition, about eight percent of the modulated genes are related to extracellular matrix (ECM) structure and dynamics (Figure 5). Expression of several genes coding basement membrane or ECM structural components, as well as proteins involved in aggrecan turnover such as *ADAMTS1* and *ADAMTS5,* was altered in TDI chondrocytes. Many genes coding enzymes involved in the glycosaminoglycan or proteoglycan biosynthesis and sulfation pathways were also modulated e.g. hyaluronan synthase 3 (*HAS3*), xylosyl-transferase (*XYLT1*), glactosyltransferases (GALNT3, *B3GALNT2*, *B4GALT5*, *GALNTL2*) glucuronyltransferases (*B3GAT2*) and glycosyltransferases (*B3GALTL*, *GLT25D2*) as well as *PAPSS2*, coding for one of the two synthetases producing *PAPS*, *SLC26A2* and several other genes encoding sulfotransferases, including *CHST3*, *HS3ST3A1, HS3ST3B1* and *HS6ST2*.

Likewise, modulated genes include genes involved in cell-cell interaction or adhesion (*CD44*, *NCAM1*, *integrins*, *cadherins* and *protocadherins*) and cell motility processes (regulation of actin cytoskeleton, myosin), such as genes that regulate or act along the Rac1 and CDC42 RhoGTPase signaling pathways. In particular, *MTSS1*, *NME1* and *RND3* were up-regulated whereas *IQGAP3* and *TIAM2* were down-regulated (Figure 5).

#### Canonical pathways and in silico promoter analysis

**Figure 6:**
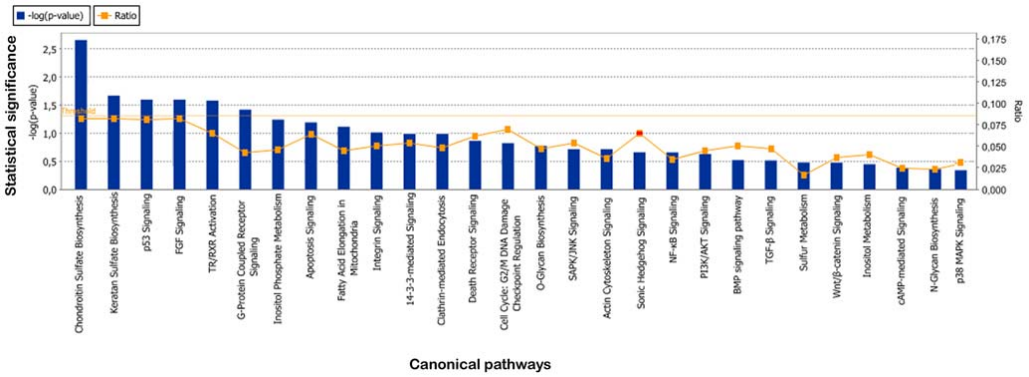
Normal v. TDI chondrocytes: canonical pathways affected by mutated FGFR3. Pathways are shown along the x axis of the classical IPA bar graph. The significance cutoff is shown as an orange horizontal line. Ratios (number of modulated gene present in a given pathway divided by the total number of genes that make up that pathway) are shown as orange points within the bar. Ratio values are shown on the right y axis.

The percentage of genes differently modulated between TDI and control chondrocytes in a given pathway was low and only two metabolic (chondroitin and keratan sulfate biosynthesis) and four signaling pathways (p53, FGF, G-protein coupled receptor signaling and thyroid hormone nuclear signaling pathways TR/RXR) were associated with significant p-values less than 5%. Despite the absence of statistically significant enrichment, many other signaling pathways may be affected as several modulated genes belong to the TGFβ/BMP, Wnt, Hedgehog, PI3K/AKT, JNK, p38 MAPK and 14-3-3 signaling pathways (Figure 6 and supplementary data S4). Moreover, some modulated genes are related to scaffold proteins, e.g. *CNKSR2, IQGAP3, LSP1* (down-regulated) and *NME1* (up-regulated).

Modulated expression was identified for 24 Transcription Factors (TFs) acting downstream of the MAPK, Wnt, Hedgehog, TGF-β, NFκB, PI3K/AKT and calcium signaling pathways (supplementary data S4). Several TFs belonging to families already known to control skeletal development were differently modulated: *JUN*, *FOSL2, CEBPD*, *ID2*, *ID3* and *ETV1*/*ETV5* were up-regulated, whereas *ATF5*, *RUNX2*, *SOX8* and *NKX3.2* were down-regulated.

Promoter analysis of modulated genes was also performed to identify over-represented TF binding sites (TFBS) in 5 kb upstream regions. About 70 TFBS were statistically over-represented using the whole genome as a reference). In contrast, no TFBS enrichment could be detected using the set of all expressed genes as reference (supplementary data S4).

**Figure 7:**
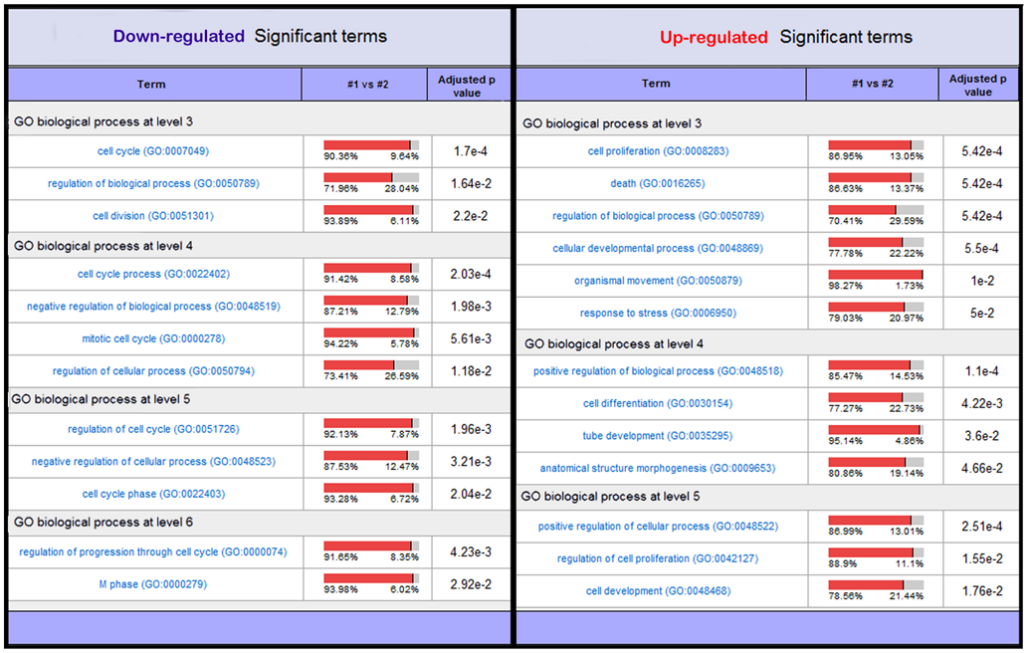
GO terms associated with up or down-regulated E2F targets. Biological processes associated with either down-regulated or up-regulated E2F targets were assessed using FATIGO+. Significance of GO term abundance was computed against expressed genes. Terms in the table are grouped by GO term levels and are sorted by adjusted p-value. The normalized percentage of genes annotated to the functional term is given in the 1 vs #2 column.

Furthermore, a search for differently modulated E2F targets identified 51 genes, of which 38% were down-regulated and 62% up-regulated. GO term enrichment analysis showed that down-regulated genes were mainly associated with cell cycle and division, whereas up-regulated genes were related to development, differentiation and death (Figure 7 and supplementary data S5).

### In Vitro Analysis

#### qPCR tests

**Figure 8:**
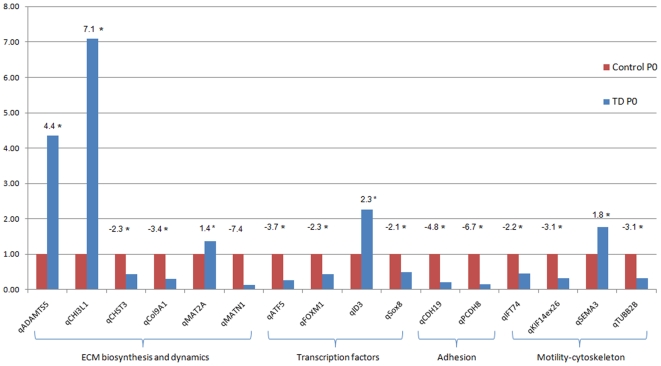
qPCR results. Relative expression levels are shown as a bar graph. Expression values were normalized to control in order to present expression ratios. Fold changes are given above each bar. Significant fold changes (5% cutoff) are indicated by an asterisk (*).

Expression of 16 genes representative of the biological processes highlighted by the IPA analysis was assessed by qPCR on three control and three pathological (Y373C) chondrocyte primary cultures (Figure 8 and supplementary data S6). Amplification efficiencies were within a range of 0.8–1.2 and no genomic DNA or negative control amplifications were observed. Fold change differences between pathological and control chondrocytes were similar to those measured by microarray. However, lower values were observed for *ID3* (2.3 instead of 3) and *SEMA3* (1.8 instead of 3). The qPCR estimated fold change for *MAT2A* was below the cutoff of 1.5 (1.4 instead of 2.4).

#### Mitosis and immunocytochemistry analysis

Mitosis in TDI and control human primary chondrocytes was assessed by counting cells at cytokinesis in primary cultures. Significant differences in the frequency of cells at cytokinesis were detected between TDI (8.3%±1.2) and control cultures (13.6%±0.9), respectively, suggesting a reduced cellular proliferation in TDI cultures (t = 1.5 10^−8^) (supplementary data S7).

**Figure 9:**
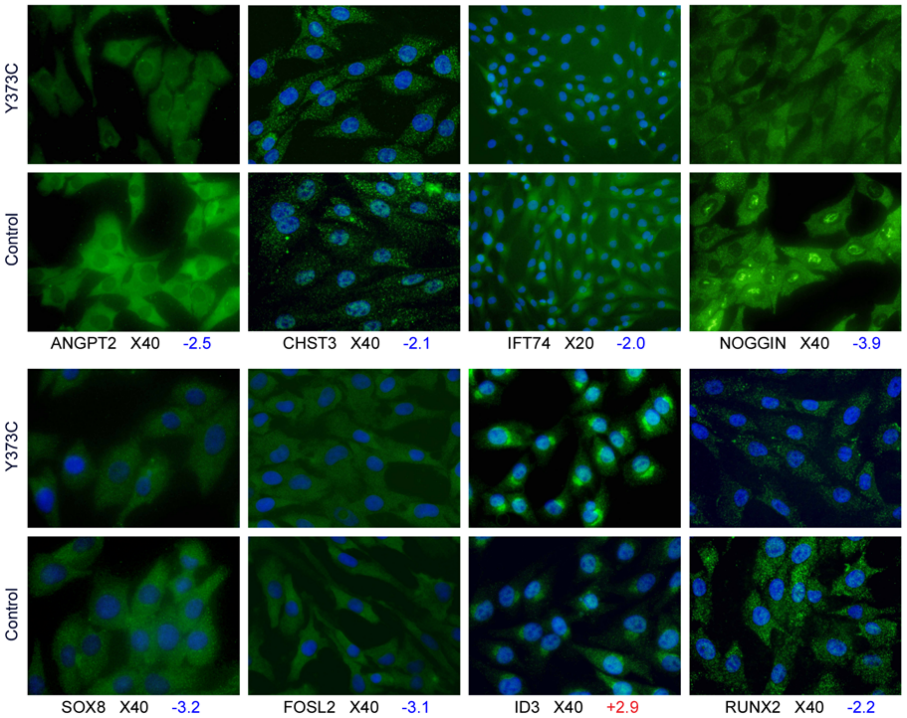
Immunocytochemistry analysis. Cytoplasmic localization and reduced staining for three transcription factors (SOX8, FOSL2, RUNX2) were observed in TDI chondrocytes (Y373C mutation). Staining for the intraflagellar transport protein (IFT74) is less marked in the mutant cells than the controls. Cytoplasmic staining for CHST3, a protein implicated in chondroitin sulfate biosynthesis, is less marked in TDI chondrocytes. Staining for ANGPT2 an NOGGIN in TDI chondrocytes is also less evident. Marked perinuclear staining of ID3 is observed in TDI chondrocytes. All the immunocytochemistry staining is in line with the fold change.

Immunocytochemistry was performed to confirm microarray results for four transcription factors (SOX8, FOSL2, ID3, RUNX2), two genes involved in microtubule dynamics (KIF2C and KIF14) as well as IFT74 (intraflagellar transport), CHST3 (chondroitin sulfate biosynthesis), Noggin (BMP signaling) and ANGPT2 (angiogenesis). Protein detection was consistent with expression data (Figure 9 and supplementary data S8). In most cases, in human primary or immortalized chondrocytes, differences in cytoplasmic protein staining were detected. However, reduced nuclear levels of CHST3 and Noggin, as well as increased perinuclear and nuclear staining of ID3 were noticed in TDI chondrocytes. Likewise, reduced staining of KIF14 was observed both in the cytoplasm and Golgi of mutant chondrocytes. In addition, no obvious modification of actin fibers was observed in the TDI chondrocytes using phalloidin and actetylated alpha-tubulin staining.

## Discussion

### Status characterization of human primary chondrocyte cultures

High levels of proliferative chondrocytic markers together with reduced or negligible expression of hypertrophic markers confirmed that the primary normal and TDI chondrocytes were not dedifferentiated and exhibited a proliferative phenotype.

### FGFR3 constitutive activation modulates cell cycle

The cell cycle is a highly coordinated process, which involves expression of a host of genes in the different cell cycle phases. Most genes essential for S, G2, and M phases, were found to be down-regulated in the human TDI primary chondrocyte cultures. Conversely, genes promoting G1 progression, especially *cyclin D1* were up-regulated, in agreement with previous studies [32], [36]. Inhibitors of G1 progression, such as *p18* were down-regulated. These data, together with the reduced frequency of chondrocytes undergoing cytokinesis observed in TDI cultures, are consistent with a decrease in TDI chondrocyte proliferation and a G1 block as observed in RCS lines upon FGF stimulation [32], [36].

Interestingly, the observed increase in cyclin D1 expression is consistent with the typical FGF mitogenic effect on most cell types [37]. Thus, the unique cell cycle inhibition response observed in TDI chondrocytes may occur during a later phase of the cell cycle, e.g. the G1/S transition as cyclin A (−2.8 fold change) can be rate limiting for S-phase entry. Alternatively, it may also result from the regulation of p107 and p130 pocket proteins, which undergo dephosphorylation upon FGF stimulation [35], [36]. *PTPRG*, a member of the protein tyrosine phosphatase family previously shown to induce pRB dephosphorylation [38], is up-regulated in TDI chondrocytes, suggesting potential involvement in this process.

Early cell cycle exit and premature chondrocyte differentiation may also be due to modulation of the ratio between activator and repressor E2Fs. In support of this hypothesis, 38% of modulated E2F targets were found to be down regulated in the current dataset, and mainly associated with the cell cycle process, whereas up-regulated E2F targets are involved in developmental and differentiation processes. Down-regulation of E2F8 in TDI chondrocytes (−2.2 fold change) may contribute to such a mechanism [39].

### FGFR3 constitutive activation alters extracellular matrix properties

Several basement membrane and ECM structural components are down-regulated in TDI chondrocytes, suggesting an altered matrix organization and turnover. A high number of modulated genes are involved in glycosaminoglycan (GAG) and proteoglycan biosynthesis, diversification and sulfation. Among these, hyaluronan synthase 3 (*HAS3*) was up-regulated. Over-expression of this gene product may result in shortened high molecular weight hydrophilic hyaluronan molecules, possibly reducing ECM viscosity [40] and cell movement throughout the cartilage. Likewise, changes in relative quantities of xylosyltransferases, glucuronyltransferases, galactosyltransferases and glycosyltransferases may also alter biochemical properties and to some extent, the function of these proteoglycans in cell adhesion, receptor activation and long-range diffusion of signaling proteins. The present data also provides an indication of a putative defect in GAG sulfation, which may lead to dramatic biological effects, as exemplified by mutations in *PAPSS2*, *SLC26A2*, *gPAPP* or *SUMF1*, all causing skeletal dysplasias [41]–[44].

### FGFR3 constitutive activation alters the cytoskeleton and adhesion

Cartilage integrity and maturation depends on well-tuned interactions with the ECM, which acts jointly with the cytoskeleton to determine chondrocyte shape and control motility [14]. In the TDI primary chondrocyte cultures, several differently modulated genes are involved in adhesion and cytoskeleton dynamics and regulate or act along the Rac1 and CDC42 RhoGTPase signaling pathways. This is in agreement with previous studies showing dwarfism, abnormal cell shape and disrupted columnar organization in mice lacking β1 integrin [9], ILK [13] and Rac1 [45].

Although functions of some of these modulated genes are somehow controversial, depending on cellular context (e.g. *ARHGDIB* can both inhibit and positively regulate *Rac1*), these observations collectively suggest that regulation of Rac1 and Rho/ROCK pathways may be impaired, possibly resulting in reduced motility. As the columnar arrangement of the proliferative zone requires chondrocytes to undergo a gliding movement with one cell moving on top of the other [46], these findings may provide clues about growth plate disorganization. The protein products of RND3, IQGAP3 and ARHGDIB, acting at the interface of ERK1/2 and RhoGTPase pathways, may be involved in the pathological process and would warrant further examination.

### FGFR3 constitutive activation disturbs the balance between multiple signaling pathways

The transcription factor binding site (TFBS) enrichment analysis suggests that TDI mutations affect almost all signaling pathways involved in chondrocyte biology. This is further supported by the modulation of 24 transcription factors that belong to, or act downstream of, the MAPK, Wnt, Hedgehog, BMP/TGF-β, NFκB, PI3K/AKT and calcium signaling pathways.

Likewise, the expression of genes coding for several key components of many signaling pathways is modified in TDI primary chondrocytes, including *DUSP6*, *MAP3K5, ZAK* and *MAP2K6* in ERK1/2 and p38MAPK signaling [47]–[49], *ID2* and *ID3* in BMP and TGFβ signaling, *PIK3R1*, *PLCG2* and *PLCB4* in inositol phosphate signaling, *FOXM1* and *MAPK8* [50] in JNK signaling, *DKK1* [51] in WNT signaling, (*HIP*) [52], *RAB23* [53], *IFT74* in hedgehog signaling or *NKX3.2* in PTHrP [18] and NFkB [54] signaling. In addition, some modulated genes code for scaffold proteins which are essential for the appropriate subcellular location of signaling molecules. For instance, down-regulation of *CNKSR2* in TDI chondrocytes may impair the crosstalk between MAPK and Ral pathways which is essential for proper receptor endocytosis, cytoskeleton remodeling and DNA synthesis [55]. Likewise, down-regulation of *IQGAP3* and *LSP1* as well as over-expression of *NME1* may modify ERK1/2 activation upon PKC or Ras activation [56]–[58].

The potentially reduced JNK signaling may lead to G1 cell cycle arrest in TDI chondrocytes, as FOXM1 (−3.1 fold change) is known to promote proliferation and control the G1/S transitions by acting through MAPK8 (−2 fold change) [50]. Simultaneously, increased BMP signaling and up-regulation of *FOSL2, EIF4A and RPS23* combined with *ATF5* and *NKX3.2* down-regulation, may induce differentiation [18], [59]–[62]. Altogether, these findings are suggestive of reduced proliferation, premature cell cycle exit and induction of differentiation.

In conclusion, signal transduction pathways represent networks that process and integrate information from the cell environment to regulate the spectrum of downstream targets in a context specific manner. This study suggests that *FGFR3* activating mutations in human chondrocytes may disturb this finely tuned process at many levels. Firstly, *FGFR3* activating mutations affect input signals (modified expression of other growth factors and membrane bound receptors, biochemical properties of the ECM). Secondly, *FGFR3* activating mutations alter the expression of several components of signaling pathways, possibly modifying the responsiveness to a number of other external stimuli. Finally, *FGFR3* activating mutations alter cytoskeleton and scaffold dynamics, resulting in improper compartmentalization and insulation. Thus, TDI pathogeny may result from the interplay of several signaling pathways and from a defective combination of signaling modules. This study provides new insight into molecular events triggered by *FGFR3* mutations during endochondral ossification. Further *in vivo* studies using compound transgenic mice will now be required to study these hypotheses.

## Materials and Methods

### Ethics Statement

All cartilage specimens in this study were consented under an IRB-approved protocol of informed consent through Hôpital Necker-Enfants Malades. All consents were written and signed by the participants in accordance with the French ethical standards.

### Chondrocyte and RNA preparation

Cartilage samples were obtained from 11 medically aborted fetuses following informed consent of parents. Chondrocytes were isolated from the growth plate as described previously [63], plated in flask (25 cm^2^) and cultured at 37°C for 4 days in DMEM supplemented with 10% fetal calf serum (Invitrogen). Chondrocytes were collected after 24 hours depletion. Total RNA was extracted using the RNeasy isolation kit (Qiagen) and treated with DNase I. Integrity and purity of total RNA were analyzed using a Bioanalyser 2100 (Agilent).

### Microarray hybridization and quality control

cDNA synthesis and labeling were performed on 3 µg of total RNA using the GeneChip one-Cycle Target Labeling and Control Reagents kit, according to the manufacturer's protocol. Human Genome U133plus2.0 GeneChips (Affymetrix) were hybridized, revealed and washed according to the Affymetrix protocol. GeneChips were scanned using a 7G scanner (Affymetrix) and images (DAT files) were converted to CEL files using GCOS software (Affymetrix). Quality Control of cRNA synthesis, hybridization and data acquisition was performed according to the manufacturer's protocol completed with personal QC and data visualization.

### Data Analysis

Data normalization and differential analyses were performed with the R-Bioconductor statistical environment [64]. After low level diagnostics with the affyQCReport package, raw data were normalized, and log_2_ intensity expression summary values for each probe set was calculated using the gcRMA algorithm. Probe sets corresponding to control genes or having a low intensity signal (median log_2_ of intensity <4) were discarded, yielding a total of 21836 probe sets for further analyses.

Preliminary analyses highlighted a strong batch effect, maybe related to changes in culture conditions having occurred during the sample collection phase. A two way ANOVA with robust variance estimation was performed with the LIMMA package [65] to evaluate effects of mutation, batch and the interaction between them. Probe sets showing significant batch and interaction effects (p-value< = 0.001) were discarded from further analysis. Modulated probe sets were then defined as having a mutation effect (p-value< = 0.05), a range_2_ > = 1.5 and a fold change > = 1.5.

Analysis of genes associated with cell functions was carried out using Ingenuity Pathways Analysis and FATIGO+ to identify biological processes and pathways which may be associated with modulated gene expression. FATIGO+, as well as whole genome rVista, were used for TFBS analysis. The Genomatix Suite was used to identify targets of E2Fs and some modulated TFs. Statistical significances were evaluated using the set of 21836 expressed probe sets as reference. All data are MIAME (minimum information about a microarray experiment)-compliant and were formatted and exported to the ArrayExpress database, according to MIAME guidelines (ArrayExpress accession number: E-MEXP-2276).

### cDNA and qPCR

cDNA were generated from 1 µg total RNA using the Verso RT PCR kit (ABgene AB-1453). RNA samples were heated at 70°C for 5 min, placed 1 min on ice before adding a pool of random hexamers and anchored oligo-dT 3∶1 (v/v) and reaction components, according to the manufacturer's protocol. Reverse transcription was performed during one hour at 50°C, followed by two minutes at 95°C.

Quantitative PCR primers were defined using the Primer Express software. Primers were chosen to generate 100–120 bp PCR products spanning at least one intron, making it possible to detect latent genomic DNA contaminations. Primer sequences are available as supplementary data (supplementary data S6).

QPCR were performed using an ABI Prism 7300 and the Absolute QPCR SYBER Green Mix (Abgene AB-1158). Reactions were set up in 25 µl, using 50 ng of cDNA and 70 nM of each primer, according to the manufacturer's protocol. A three step cycling method was used (95°C 15 s, 60–62°C 15 s, 72°C 30 s) for 40 cycles and melt curves were analyzed to confirm the specificity of the reaction.

Standard samples (3 quantity obtained by 1/100 serial dilution) were added to measure amplification efficiencies and all experiments were carried out in triplicate on the same cDNA preparations. Quality control and relative quantity calculations were performed using the qBase software [66]. Normalization was performed with two reference genes in order to improve result accuracy and reliability. *RPLP13A*, *TBP*, *ACTB* and 18S primer sets were evaluated as potential control transcripts, based on previous results [67] and *ACTB* and *RPL13* were finally chosen as endogenous control genes.

### Mitosis and Immunocytochemistry analysis

Primary and immortalized chondrocytes [68] were seeded in 8 wells Lab-Tek chamber slides NUNC field with DMEM+10% fetal calf serum (Invitrogen) in 5% CO_2_ at 37°C. Three different controls and four TDI cultures (Arg248Cys or Tyr373Cys mutations) were cultured on each slide, leaving one empty negative control chamber. Each slide was then analyzed with a different antibody. This design ensured that all samples were subjected to the same conditions, thus reducing experimental bias.

For mitosis analysis, three control and three TDI primary cultures stained by DAPI were analyzed. A total of 1200 cells in 10 random fields were counted for each category.

For immunocytochemistry, cells were fixed in 4% PFA for 20 min, permeabilized in 0.1% TritonX-100/PBS and blocked in 10% sheep or donkey serum, depending on primary antibody. Immunocytochemistry was performed as previously described [69], using Alexa488 or Alexa568 (Molecular Probes) secondary antibodies. Primary antibody references and working dilutions are provided as supplementary data S8. No background was detectable in empty wells and when the primary antibody was omitted. Cells were covered with mounting solution (Vector), analyzed field by field and examined using an Olympus IX2-UCB microscope using 20x and 40x objectives.

Immunofluorescence pictures for a given antibody were selected as good representatives of the samples and were taken using the same camera settings in order to allow semi-quantitative signal comparisons.

## Supporting Information

Supplementary data S1 (0.02 MB XLS)

Supplementary data S2 (2.16 MB XLS)

Supplementary data S3 (1.25 MB XLS)

Supplementary data S4 (0.22 MB XLS)

Supplementary data S5 (0.02 MB XLS)

Supplementary data S6 (0.32 MB XLS)

Supplementary data S7 (1.55 MB XLS)

Supplementary data S8 (3.27 MB XLS)
